# A Comparative Dosimetric Analysis of Volumetric Modulated Arc Therapy (VMAT) Versus Intensity-Modulated Radiation Therapy (IMRT) for Head and Neck Cancer in an Indian Population

**DOI:** 10.7759/cureus.100283

**Published:** 2025-12-28

**Authors:** Nikhil Kumar Athmakoor, Sandesh Potaraju, Aarathi Ardha, Tangallapally Nikhitha, Srinivas Vuppu, B. Ravi Kumar, Ramanjaneyulu U.G.

**Affiliations:** 1 Radiation Oncology, Mahatma Gandhi Memorial Hospital, Warangal, IND; 2 Radiation Oncology, MNJ Institute of Oncology and Regional Cancer Centre, Osmania Medical College, Hyderabad, IND; 3 Medical Physics, MNJ Institute of Oncology and Regional Cancer Centre, Osmania Medical College, Hyderabad, IND; 4 Medical Physics, Government Siddhartha Medical College, Vijayawada, IND

**Keywords:** dosimetry, head and neck cancer, imrt, radiotherapy, treatment planning, vmat

## Abstract

Background

Radiotherapy is a primary treatment for head and neck cancers (HNC). Intensity-modulated radiation therapy (IMRT) is the standard, but volumetric modulated arc therapy (VMAT) has emerged as a faster alternative. This study compares the dosimetric and planning parameters of IMRT and VMAT for HNC patients in an Indian population.

Aims

To prospectively compare IMRT and VMAT plans for HNC patients, focusing on planning target volume (PTV) coverage, dose to organs at risk (OARs), conformity index (CI), homogeneity index (HI), monitor units (MUs), and treatment time.

Methods

This prospective observational study included 30 patients with HNC. For each patient, both a seven-field sliding window IMRT plan and a double-arc VMAT plan were generated. Dosimetric parameters for PTVs (66 Gy, 60 Gy, and 54 Gy) and OARs (spinal cord, brainstem, mandible, and parotids) were compared using paired statistical tests.

Results

Both techniques produced clinically acceptable plans. IMRT plans demonstrated statistically superior PTV coverage, with better D95% and D98% values across all target volumes (p < 0.01 for PTV66 D95% and D98%). IMRT also yielded slightly better conformity for the PTV66 (CI 95%: 0.98 vs. 0.97 for VMAT, p < 0.01). VMAT plans provided significantly better sparing of the parotid glands (p ≤ 0.01). While IMRT resulted in a lower maximum dose to the brainstem (40.88 Gy vs. 48.74 Gy, p < 0.01), doses from both plans were within tolerance. VMAT plans were significantly more efficient, reducing the mean number of MUs by 58.5% (1342.79 vs. 556.50) and the mean treatment time by 58.6% (3.67 min vs. 1.52 min) (p < 0.01 for both).

Conclusion

VMAT provides comparable, clinically acceptable plans to IMRT while offering superior parotid sparing and a significant reduction in treatment time and MUs. These efficiencies make VMAT a highly advantageous technique, particularly in high-volume cancer centres.

## Introduction

Head and neck cancers (HNC) arise from the lip, oral cavity, pharynx, larynx, salivary glands, paranasal sinuses, and nasal cavity. The absolute incidence worldwide crossed 900,000 in 2022, with Asia having the highest share, constituting 63.9%. India has the greatest HNC burden, with 26.2% of the worldwide incidence, and also has the highest mortality rate, at 28.6% [1]. In India, HNC constitutes a significant health problem, accounting for approximately one-third of all cancer cases, compared to 4%-5% in developed nations [2]. While smoked tobacco and alcohol are major global risk factors, in the Indian population, smokeless tobacco, betel nut, and Epstein-Barr virus play a vital role [3].

Radiotherapy is a cornerstone of treatment for HNC, used both as a primary modality and as an adjuvant therapy [4]. The transition from 3D conformal radiotherapy (3D-CRT) to intensity-modulated radiation therapy (IMRT) has made treatment safer and more beneficial, establishing IMRT as the gold standard for its superior dose conformity and sparing of organs at risk (OARs), such as the parotid glands, spinal cord, and brainstem [5,6].

However, IMRT has disadvantages, including increased treatment delivery time and a higher number of monitor units (MUs) [7,8]. Volumetric modulated arc therapy (VMAT), a newer rotational technique, was developed to address these drawbacks. VMAT allows for variations in gantry speed, dose rate, and collimator angle, potentially reducing treatment time, MUs, and the risk of secondary malignancies, while maintaining or improving dose distribution [7-10].

This study aims to compare IMRT and VMAT regarding planning and dosimetry in HNC within this specific demographic to enhance evidence-based therapeutic decision-making.

## Materials and methods

Study design and population

A prospective observational study was conducted at the Department of Radiotherapy, MNJ Institute of Oncology and Regional Cancer Centre, Hyderabad, India, between June 2017 and June 2018 (approval no. 16107001113D). The study population comprised patients with head and neck malignancies eligible for treatment with IMRT or VMAT. All eligible subjects who were willing to participate were consecutively sampled.

Sample size

The sample size was calculated based on a study by Vanetti et al. [11], assuming an expected mean target homogeneity of 9.9 (SD 1.06) in the IMRT group and 8.8 (SD 2.04) in the VMAT group, with 80% power and 5% alpha error. The minimum required sample size was calculated to be 29 subjects; thus, 30 subjects were included in the final analysis.

Patient selection and pre-treatment evaluation

For inclusion in this study, patients were required to have a histopathologically confirmed diagnosis of invasive squamous cell carcinoma located in the oropharynx, hypopharynx, larynx, or oral cavity. The tumour had to be classified as Stage I-IV according to the TNM classification system, and patients needed to be over 20 years of age, with a performance status of 0-2 based on the Eastern Cooperative Oncology Group (ECOG) criteria. Additionally, participants were required to have normal complete blood count (CBC) results, normal liver and kidney function tests, and provide informed consent.

Patients were excluded from the study if they presented with distant metastases or had a history of previous surgical excision (excluding biopsy) or irradiation to the head and neck region. Other exclusion criteria included a planned elective surgery, the presence of synchronous multiple malignancies, a prior history of HNC, or evidence of mandibular invasion.

Before treatment, every patient underwent a thorough evaluation. This included a detailed medical history and a complete physical examination, during which height, weight, body surface area, and performance status were documented. The pre-treatment workup also required histopathologic proof of squamous cell carcinoma, a CBC with differential and platelet counts, and tests for blood urea, serum creatinine, random blood sugar, liver function, and viral markers. To complete the staging and evaluation process, all patients had a computed tomography (CT) scan of the neck and chest, and underwent a comprehensive oral and dental evaluation to ensure proper care before radiotherapy.

Radiotherapy technique and quality assurance (QA)

All patients were treated using the VMAT simultaneous integrated boost technique. The delineation of all tumour volumes, nodal volumes, and OARs was performed in accordance with the Radiation Therapy Oncology Group (RTOG) contouring guidelines, while International Commission on Radiation Units and Measurements (ICRU) reports 50, 62, and 83 were used to define the specific tumour volumes.

To ensure accuracy, all treatment plans underwent rigorous QA. Absolute dosimetry was conducted for every plan, which involved dose verification at the isocentre using a PMMA slab phantom (IBA Dosimetry, Schwarzenbruck, Germany) and a 0.6 cc FARMER TYPE chamber for all measurements. For relative dosimetry, pre-treatment QA involved fluence measurements, which were performed for all plans using either the electronic portal imaging device (EPID) or an IMatriXX device (IBA Dosimetry). The measured fluences were then compared with the fluences calculated by the ECLIPSE treatment planning system. This comparison utilised a gamma evaluation, with parameters set at a 3 mm distance-to-agreement and a 3% dose difference for the fluence analysis.

Treatment protocol and planning

The treatment process began after informed consent was obtained, with the study protocol being explained in detail to the patient and their attendants in their native language. For treatment, each patient was immobilised in a supine position using a thermoplastic facial mask. The neck was slightly extended, and an appropriate headrest was used to ensure comfort and a reproducible daily setup, while the hands were placed by the patient's side to minimise motion. The patient was then aligned using laser beams before proceeding to CT simulation and image acquisition. A planning CT scan, both with and without IV contrast, was performed from the vertex to the mid-thoracic region, with a slice thickness of 3 mm, on a Philips Bigbore 16-slice CT simulator (Philips, Amsterdam, the Netherlands). Orthogonal room lasers were used to place skin markers to verify that no shift occurred between scans, and the images were subsequently transferred to the ECLIPSE treatment planning system.

During the planning phase, several structure sets were contoured. The gross tumour volume (GTV) was defined as the visible tumour and any enlarged or suspicious lymph nodes identified clinically or radiographically, and was subdivided into GTVP for the primary tumour and GTVN for the nodal volume. The clinical target volume (CTV), representing tissues at risk of microscopic disease, was divided into three levels. The high-risk CTV (CTVHR) included the GTV plus a 5 mm margin, respecting anatomical boundaries. The intermediate-risk CTV (CTVIR) encompassed the GTVP with a 1-2 cm margin, including areas of potential microscopic spread. The low-risk CTV (CTVLR) included all neck nodal regions not covered by the CTVHR or CTVIR, contoured according to consensus guidelines from the Danish Head and Neck Cancer Group (DAHANCA), the European Organisation for Research and Treatment of Cancer (EORTC), and other groups. Planning target volumes (PTVs) for each risk level were generated by adding a uniform 5 mm margin to the corresponding CTV. OARs, including both parotid glands, the mandible, the glottic larynx, the spinal cord, and the brainstem, were also contoured.

The prescribed radiation doses were delivered in 30 fractions. The PTVHR received 66 Gy (220 cGy per fraction), the PTVIR received 60 Gy (200 cGy per fraction), and the PTVLR received 54 Gy (180 cGy per fraction). The primary objective for target coverage was to ensure that at least 99% of the PTV volume received 95% of the prescribed dose, with strict limits on dose heterogeneity: no more than 2% of the PTV receiving over 110%, and no more than 1% receiving less than 93% of the prescribed dose. For critical structures, the first priority was to keep spinal cord and brainstem doses below their tolerance limits. The second priority was to reduce the mean dose to the parotid glands to below 26 Gy, where possible. Dose constraints for OARs were a maximum of 50 Gy for the spinal cord, less than 54 Gy for the brainstem, a mean dose of less than 25 Gy to both parotids, and a maximum of 70 Gy to the mandible.

Treatment planning was performed using the ECLIPSE system with the anisotropic analytical algorithm. IMRT plans were generated with a seven-field sliding window technique, while VMAT plans consisted of two coplanar arcs with a rotated collimator to ensure full tumour coverage. All plans were evaluated using isodose lines and dose-volume histograms (DVHs) before approval and delivery on a Varian TrueBeam linear accelerator (Varian Medical Systems, Inc., Palo Alto, CA, USA). Parameters analysed from the DVHs included the homogeneity index (HI), conformity index (CI), and target homogeneity for the PTV, as well as maximum doses to the spinal cord, mandible, and brainstem, and the mean dose to the parotids. HI was evaluated as per ICRU 83, i.e., D2%-D98%/D50% (ratio of the difference between the dose covering 2% and 98% to the dose received by 50% of the PTV volume in Gy). Smaller values represent increasing dose homogeneity, with the value of 0 being ideal. CI 95% is defined as the ratio between the patient volume receiving at least 95% of the prescribed dose and the volume of PTV (in cubic cm). Ideally, it should lie between 0.9 and 2.0. Target homogeneity was expressed by (D5%-D95%), defined as the difference between the dose covering 5% and 95% of the PTV (in cubic cm). Total MUs were recorded after plan evaluation.

In addition to radiotherapy, eligible patients received concurrent chemotherapy, with weekly cisplatin at a dose of 40 mg/m² for four to six cycles.

Objectives and outcome measures

The primary objective was to compare the planning and dosimetric parameters between IMRT and VMAT in HNC. Secondary objectives included comparing the cumulative DVHs for the target and OARs (mandible, brainstem, spinal cord, and parotid glands), as well as comparing the HI, CI, and total MUs.

Dosimetric parameters such as D98%, D2%, D95%, and D5% for the PTVs were analysed. The maximum doses to OARs and the mean doses to the parotid glands were also reported for both IMRT and VMAT plans.

Statistical analysis

Statistical analysis was performed using IBM SPSS Statistics for Windows, Version 20 (Released 2011; IBM Corp., Armonk, NY, USA). Paired Samples t-test and Wilcoxon Signed Ranks Test were used to compare the dosimetric parameters between the two techniques. A p-value of <0.05 was considered statistically significant.

## Results

A total of 30 subjects were included in the final analysis. The study population consisted of 21 males (70%) and 9 females (30%). The mean age was 53.73 ± 11.98 years. The most common tumour site was the oral cavity (56.67%), and the majority of patients presented with advanced disease (Stage IVA or higher, in 70% of cases). The predominant histology was well-differentiated squamous cell carcinoma (43.33%), while others had moderate and poor differentiation.

Target volume dosimetry (PTV)

PTV 66 Gy

IMRT plans showed statistically significantly better coverage for the high-dose PTV. The mean D95% was 64.5 Gy for IMRT versus 63.5 Gy for VMAT (p < 0.01), and the mean D98% was 63.3 Gy for IMRT versus 62.4 Gy for VMAT (p < 0.01). The CI 95% was also significantly better for IMRT (0.98 vs. 0.97, p < 0.01). However, the HI was similar between the two techniques (p = 0.06) (Table 1).

**Table 1:** Comparison of dosimetric parameters of PTV 66 in IMRT and VMAT ^a ^Paired Samples Test; ^b^ Wilcoxon Signed Ranks Test; NA, not applicable; Z - standardised statistic; t - t-test statistic * Means of all the parameters were compared, and appropriate statistical tests were used PTV, Planning Target Volume; IMRT, Intensity-Modulated Radiation Therapy; VMAT, Volumetric Modulated Arc Therapy; TH, Target Homogeneity; HI, Homogeneity Index; CI, Conformity Index; SD, Standard Deviation

	IMRT				VMAT					
PTV 66	Mean (±SD)	Median	Min	Max	Mean (±SD)	Median	Min	Max	Test statistic	p-value^*^
Volume (median cm^3^)	220.1 (160.3)	183.1	47.3	884.5	220.1 (160.3)	183.1	47.3	884.5	NA	NA
Mean Dose % (Gy)	101.6 (0.9)	101.8	99.6	103.3	100.7 (1.2)	100.4	99.3	103.2	-3.09 (Z)	<0.01^b^
D2% (Gy)	69.2 (0.8)	69.1	67.1	71.2	69 (1.1)	68.9	67.3	71.7	0.88 (t)	0.38^a^
D5% (Gy)	68.8 (0.8)	68.8	66.9	70.6	68.6 (1.0)	68.4	66.9	71.6	1.16 (t)	0.24^a^
D95% (Gy)	64.5 (0.8)	64.5	62.5	65.9	63.5 (0.9)	63.6	61.6	65.2	6.00 (t)	<0.01^a^
TH (D5%-D95%) (Gy)	4.3 (1.0)	4.1	2.9	7.3	5.0 (1.3)	5	2.6	9	-2.43 (t)	0.02^a^
D98% (Gy)	63.3 (1.3)	63.6	58.8	65.2	62.4 (1.5)	62.7	58.8	66.1	-3.09 (Z)	<0.01^b^
CI95%	0.98 (0.01)	0.98	0.95	1	0.97 (0.02)	0.97	0.91	1	-3.84 (Z)	<0.01^b^
HI	0.087 (0.02)	0.079	0.06	0.17	0.098 (0.03)	0.1	0.05	0.18	-1.82 (Z)	0.06^b^

PTV 60 Gy and PTV 54 Gy

Similar trends were observed for the intermediate and low-dose PTVs. IMRT consistently provided statistically superior D95% and D98% values (p < 0.01 for PTV60; p = 0.03 and p = 0.01 for PTV54, respectively). Other parameters, like D2%, D5%, and HI, were not significantly different between the two plans for these volumes (Tables 2, 3).

**Table 2:** Comparison of dosimetric parameters of PTV 60 in IMRT and VMAT ^a^ Paired Samples Test; ^b^ Wilcoxon Signed Ranks Test; NA, not applicable; Z - standardised statistic; t - t-test statistic * Means of all the parameters were compared, and appropriate statistical tests were used PTV, Planning Target Volume; IMRT, Intensity-Modulated Radiation Therapy; VMAT, Volumetric Modulated Arc Therapy; TH, Target Homogeneity; HI, Homogeneity Index; CI, Conformity Index; SD, Standard Deviation

	IMRT				VMAT					
PTV 60	Mean (±SD)	Median	Min	Max	Mean (±SD)	Median	Min	Max	Test statistic	p-value*
Volume (median cm^3^)	315.1 (111.3)	307.1	75	520.1	315.1 (111.3)	307.1	75	520.1	NA	NA
Mean Dose %(Gy)	93.1 (0.94)	93.3	91	95	92.3 (1.27)	91.85	90.2	94.5	-2.88 (Z)	<0.01^b^
D2% (Gy)	64.84 (0.97)	65.08	62.13	66.72	64.35 (1.28)	64.17	62.65	66.84	1.87 (t)	0.07^a^
D5% (Gy)	64.11 (0.9)	64.36	61.44	65.86	63.73 (1.23)	63.44	62.13	66.02	-1.56 (Z)	0.11^b^
D95% (Gy)	58.1 (1.27)	58.16	54.95	59.97	57.33 (0.75)	57.4	55.29	58.67	3.49 (t)	<0.01^a^
TH (D5%-D95%) (Gy)	6.01 (1.5)	6.09	3.15	9.29	6.4 (1.3)	6.27	4	8.88	-1.22 (t)	0.23^a^
D98% (Gy)	56.7 (1.85)	57.18	52.17	59.26	55.87 (1.02)	55.98	53.44	57.5	2.84 (t)	<0.01^a^
HI	0.132 (0.034)	0.131	0.08	0.21	0.139 (0.027)	0.135	0.09	0.19	-1.07 (t)	0.29^a^

**Table 3:** Comparison of dosimetric parameters of PTV 54 in IMRT and VMAT ^a^ Paired Samples Test; ^b^ Wilcoxon Signed Ranks Test; NA, not applicable; Z - standardised statistic; t - t-test statistic * Means of all the parameters were compared, and appropriate statistical tests were used PTV, Planning Target Volume; IMRT, Intensity-Modulated Radiation Therapy; VMAT, Volumetric Modulated Arc Therapy; TH, Target Homogeneity; HI, Homogeneity Index; CI, Conformity Index; SD, Standard Deviation

	IMRT				VMAT					
PTV 54	Mean (±SD)	Median	Min	Max	Mean (±SD)	Median	Min	Max	Test statistic	p-value*
Volume (median cm^3^)	131.9 (63.3)	113.5	37.6	306.1	131.9 (63.3)	113.5	37.6	306.1	NA	NA
Mean Dose % (Gy)	83.6 (0.9)	83.6	82.1	85.4	83.1 (1.1)	82.6	81.8	85.3	-2.52 (Z)	0.01^b^
D2% (Gy)	58.14 (1.36)	57.69	55.53	62.66	57.92 (1.26)	57.65	56.05	60.31	-0.91 (Z)	0.36^b^
D5% (Gy)	57.2 (1.18)	57.18	53.89	59.62	57.15 (1.09)	56.97	55.53	59.11	0.21 (t)	0.82^a^
D95% (Gy)	52.85 (0.98)	52.98	50.91	54.55	52.41 (1.0)	52.34	50.12	55.56	2.17 (t)	0.03^a^
TH (D5%-D95%) (Gy)	4.35 (1.5)	4.13	1.58	7.6	4.73 (1.23)	4.77	2.15	6.95	-1.36 (t)	0.18^a^
D98% (Gy)	51.80 (1.19)	51.81	49.57	53.49	51.15 (1.02)	51.11	48.63	53.35	2.70 (t)	0.01^a^
HI	0.11 (0.03)	0.12	0.04	0.2	0.12 (0.02)	0.12	0.05	0.18	-1.20 (t)	0.23^a^

Organs at risk (OARs) sparing

VMAT plans demonstrated significantly better sparing of the parotid glands. The mean dose to the right parotid was 28.91 Gy with VMAT compared to 30.01 Gy with IMRT (p < 0.01). Similarly, the mean dose to the left parotid was 32.73 Gy with VMAT versus 33.91 Gy with IMRT (p = 0.01).

For the brainstem, IMRT plans resulted in a significantly lower maximum dose (40.88 Gy) compared to VMAT plans (48.74 Gy) (p < 0.01), although both were within the tolerance limit of 54 Gy. The maximum doses to the spinal cord and mandible were not significantly different between the two techniques (p = 0.05 and p = 0.14, respectively) (Table 4).

**Table 4:** Comparison of OAR doses, MUs and treatment times in IMRT and VMAT ^b^ Wilcoxon Signed Ranks Test; Z - standardised statistic * Means of all the parameters were compared, and appropriate statistical tests were used OAR, Organ at Risk; MU, Monitor Units; IMRT, Intensity-Modulated Radiation Therapy; VMAT, Volumetric Modulated Arc Therapy; SD, Standard Deviation

	IMRT				VMAT					
OARs	Mean (±SD)	Median	Min	Max	Mean (±SD)	Median	Min	Max	Test statistic (Z)	p-value*
Spinal cord (Dmax) (Gy)	43.39 (3.66)	44.72	34.42	49.32	44.43 (4.38)	45.77	34	49.16	-1.93	0.05^b^
Brainstem (Dmax) (Gy)	40.88 (8.09)	41.27	22.06	55.9	48.74 (7.9)	49.99	18.5	57.35	-4.40	<0.01^b^
Mandible (Dmax) (Gy)	69.34 (3.42)	69.83	56.25	73.5	68.83 (4.08)	69.97	53.18	72.72	-1.47	0.14^b^
Right parotid (Mean) (Gy)	30.01 (10.36)	27.57	19.38	61.52	28.91 (10.14)	26.52	16.54	58.62	-2.81	<0.01^b^
Left parotid (Mean) (Gy)	33.91 (12.41)	30.26	18.06	59.95	32.73 (12.01)	30.77	13.8	55.78	-2.53	0.01^b^
MUs	1342.79 (244.77)	1310.15	799.70	2030.60	556.50 (142.61)	548.80	364.40	883.90	-4.78	<0.01^b^

Treatment efficiency

VMAT plans were markedly more efficient. The mean number of MUs was significantly lower with VMAT (556.50) compared to IMRT (1342.79), representing a 58.5% reduction (p < 0.01). This translated to a significantly shorter mean treatment time for VMAT (1.52 minutes) compared to IMRT (3.67 minutes), a reduction of 58.6% (p < 0.01).

## Discussion

HNC is one of the major threats to public health in the developed world and increasingly in the developing world [1]. The increase in incidence of HNC cases is a cause of major concern, as it is associated with high morbidity and mortality. Evolution of radiotherapy from conventional to intensity modulated radiotherapy and a novel approach, volumetric modulated radiation therapy, is making the future hopeful. IMRT has been the most widely used radiotherapy method across the globe, but it was associated with many disadvantages. To avoid drawbacks offered by IMRT, a new approach, VMAT or RapidArc, has evolved, which was reported to offer faster delivery times, use fewer MU, and potentially decrease the risk of secondary malignancies, and to have superior dose distributions compared to clinical IMRT plans [7,8]. Available literature consists of many comparative studies of IMRT and VMAT treatment plans for HNC, but such studies in the Indian population are few.

This study provides a dosimetric comparison between IMRT and VMAT for HNC in an Indian population, where the primary tumour site was predominantly the oral cavity, unlike many Western studies that focus on oropharyngeal and hypopharyngeal cancers. The majority (43.33%) of subjects had stage T4a, followed by T2 (33.33%).

Our findings indicate that while both techniques produce clinically acceptable plans, IMRT provided statistically superior target coverage in terms of D95% and D98% and better conformity for the highest dose volume (PTV66). This is in contrast to some studies, where VMAT showed better or equivalent target coverage [11]. This could be attributed to the learning curve associated with the newer VMAT technique at our institution or the lateralised nature of oral cavity tumours, which may be more optimally treated with fixed-gantry IMRT fields.

Regarding OAR sparing, VMAT was superior in reducing the mean dose to both parotid glands, which is consistent with findings from several other studies, and is critical for reducing xerostomia-related quality of life issues [11-14]. Interestingly, IMRT plans achieved a lower maximum dose to the brainstem. This may be due to the anterior location of most tumours in our cohort, where fixed IMRT fields could better avoid exit doses through the brainstem, compared to the rotational delivery of VMAT.

The most significant advantage of VMAT observed in this study was its efficiency. The substantial reduction in both MUs (58.5%) and treatment time (58.6%) aligns with the bulk of published literature [11,15-26]. This efficiency is particularly valuable in high-volume centres, as it improves patient throughput, enhances patient comfort, and reduces the potential for intrafractional motion. The reduction in MUs also corresponds to a lower peripheral dose, which may decrease the risk of radiation-induced secondary malignancies.

Limitations

This was a single-institution study, so the findings may not be generalisable. The study was not powered to assess the clinical outcomes of tumour control or toxicity, nor could it correlate tumour site with dosimetric parameters.

## Conclusions

Both IMRT and VMAT planning techniques produced dosimetrically acceptable plans for HNC. IMRT demonstrated slightly better target coverage and conformity. However, VMAT provided superior sparing of the parotid glands and resulted in a significant reduction in MUs and treatment time. Given these efficiencies, VMAT is a highly advantageous and recommended technique, particularly for high-volume cancer centres, as it can significantly improve clinical workflow without compromising plan quality. Further studies are warranted to assess the long-term clinical outcomes.
